# Thyroid Hormone Availability and Action during Brain Development in Rodents

**DOI:** 10.3389/fncel.2017.00240

**Published:** 2017-08-14

**Authors:** Soledad Bárez-López, Ana Guadaño-Ferraz

**Affiliations:** ^1^Department of Endocrine and Nervous System Pathophysiology, Instituto de Investigaciones Biomédicas Alberto Sols, Consejo Superior de Investigaciones Científicas (CSIC), Universidad Autónoma de Madrid (UAM) Madrid, Spain; ^2^Center for Biomedical Research on Rare Diseases (Ciberer), Instituto de Salud Carlos III Madrid, Spain

**Keywords:** thyroid hormones, brain, rodents, type 2 deiodinase, MCT8, neurodevelopment, brain barriers

## Abstract

Thyroid hormones (THs) play an essential role in the development of all vertebrates; in particular adequate TH content is crucial for proper neurodevelopment. TH availability and action in the brain are precisely regulated by several mechanisms, including the secretion of THs by the thyroid gland, the transport of THs to the brain and neural cells, THs activation and inactivation by the metabolic enzymes deiodinases and, in the fetus, transplacental passage of maternal THs. Although these mechanisms have been extensively studied in rats, in the last decade, models of genetically modified mice have been more frequently used to understand the role of the main proteins involved in TH signaling in health and disease. Despite this, there is little knowledge about the mechanisms underlying THs availability in the mouse brain. This mini-review article gathers information from findings in rats, and the latest findings in mice regarding the ontogeny of TH action and the sources of THs to the brain, with special focus on neurodevelopmental stages. Unraveling TH economy and action in the mouse brain may help to better understand the physiology and pathophysiology of TH signaling in brain and may contribute to addressing the neurological alterations due to hypo and hyperthyroidism and TH resistance syndromes.

## Introduction

Thyroid hormones (THs) are essential for the correct development of vertebrates controlling cell growth and metabolism in all tissues. The developing CNS is particularly sensitive to impairments in TH signaling. It is well known that TH deficit leads to numerous alterations the severity of which depends on the timing and the cause of the hormonal deficit (Morreale de Escobar et al., 2004; Zoeller and Rovet, 2004). While the wide range of clinical manifestations of adult onset hypothyroidism are usually reversed with an appropriate treatment, THs deficit during development can lead to irreversible neurological alterations unless treated with a timely initiation of hormonal replacement therapy (Morreale de Escobar et al., 2000), highlighting the importance of both appropriate levels of THs and timing of action.

THs are synthesized in the thyroid gland. Out of the total hormonal secretion of the thyroid gland approximately 93% is as thyroxine or 3,5,3′,5′-tetraiodo-L-thyronine (T4) and 7% as 3,5,3′-triodo-L-thyronine (T3) which is the active nuclear form (Bernal, 2015). The main pathway of THs action is at the genomic level by regulating gene expression through the binding of T3 to nuclear TH receptors (TRs) which function as ligand-modulated transcription factors (Cheng et al., 2010). In the brain deiodinases (D2 and D3) play an essential role in maintaining appropriate levels of T3. D2 catalyzes the conversion of T4 into the genomically active T3 while D3 catalyzes the deiodination of T4 and T3 into 3,3′5′-triiodo-L-thyronine (rT3) and 3,3′-diiodo-L­thyronine (T2) respectively, inactivating TH action (Bianco and Kim, 2006). Another crucial step for TH action is the transport of THs across the brain barriers and the plasma membrane of target cells by TH membrane transporter proteins. Among the many TH transporters that have been shown to mediate TH transport, the monocarboxylate transporter 8 (MCT8) presents the highest specificity for THs. Mutations in this transporter lead to the Allan-Herndon-Dudley syndrome with severe endocrine and neurological alterations (Visser, 2007; Fu et al., 2013; Bernal, 2015). In view of this, several regulatory mechanisms fine-tune the brain content of T4 and T3. These include TH secretion by the thyroid gland, TH transport to the brain and neural cells, activity of deiodinases, and in the fetus, transplacental passage of maternal THs (Obregon et al., 1984; Morreale de Escobar et al., 1990).

Most of the knowledge regarding TH action in the developing brain has arisen from studies in rats. However, due to the advantages of using genetic engineering approaches to generate genetically modified animals in mice over rat, the mouse has become the most widely used model to study thyroid physiology and TH action in brain. The use of genetically modified mice is providing new insights into thyroid physiological and pathological events. Despite this, many events underlying TH availability in the brain during mouse fetal development still need to be unraveled in order to fully understand TH economy in the developing brain. The aim of this review is to give an up-to-date insight into the evidence that has led to the understanding of TH availability and action in the developing brain of both rats and mice especially during embryonic stages.

## Ontogeny of Thyroid Hormone Action

In the rat, tissues from whole embryos are provided with small amounts of T4 and T3 from at least embryonic day 11 (E11; Obregon et al., 1984), well before the onset of fetal thyroid gland function, which starts after E17. As the fetal thyroid gland starts functioning, the proportion of fetal THs increases so at term around 17.5% of the fetal pool of T4 (Morreale de Escobar et al., 1990) and 47% of the fetal pool of T3 are of maternal origin (Grijota-Martínez et al., 2011). In the mouse, the contribution of maternal T4 at term is larger than in rats as 60% of the total TH content in peripheral tissues derive directly from the mother (Bárez-López et al., 2017b).

**Figure 1:**
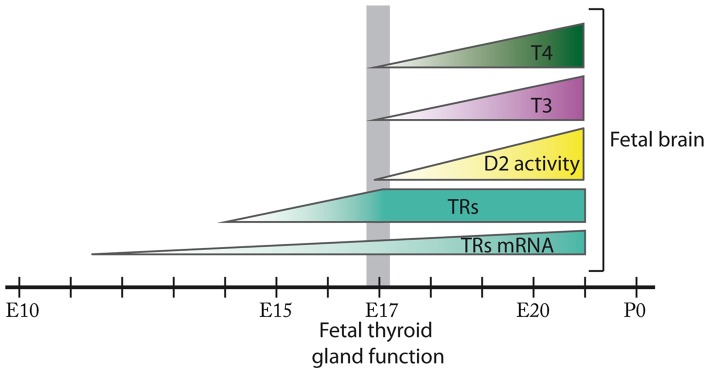
Ontogenesis of thyroid hormone action during fetal brain development in the rat. In the rat brain, thyroid hormone receptors (TRs) mRNA expression is detected from E11.5 and TRs from E14. D2 activity in addition to 3,5,3′,5′-tetraiodo-L-thyronine (T4) and 3,5,3′-triodo-L-thyronine (T3) content are present in the rat fetal brain from at least E17 when the onset of fetal thyroid function takes place. D2 activity, T4 and T3 content progressively increase in the rat fetal brain from E17 until E21. The size of the figures does not represent relative abundance. Most of these events remain to be fully elucidated in the mouse.

The few studies reporting T3 and T4 content in the developing rat brain have been restricted to periods coinciding with or following the onset of fetal thyroid function at E17–E21 (Morreale de Escobar et al., 1985; Ruiz De Oña et al., 1988; Figure 1). In mice, T4 content in brain has been detected from at least E16 (Dong et al., 2015) which is just before the onset of the fetal thyroid gland that in mice starts around E16.5 (Fernández et al., 2015), and T3 has been detected from at least E18 (Ferrara et al., 2013; Bárez-López et al., 2017b).

In the rat, mRNA for TRs has been detected from E11.5 at the neural tube and from E12.5 in certain areas of the prosencephalon, mesencephalon and the rhombencephalon (Bradley et al., 1992). In addition, T3 binding assays have revealed the presence of TRs in brain from E14, preceding the development of thyroid function, and their concentration has been shown to increase progressively until E17 (Perez-Castillo et al., 1985). Furthermore, at least from E21 there is a T3 receptor occupancy of 38% (Ferreiro et al., 1990; Figure 1). To our knowledge there are no studies describing the expression of TRs in the fetal mouse brain.

In the rat, deiodinase activity assays have detected D2 in the brain from as early as E17 with increasing activity until the day prior to birth (Ruiz De Oña et al., 1991; Figure 1). In the mouse, *Dio2* mRNA expression has been detected in the brain from at least E15 (Bárez-López et al., 2017b), suggesting that maternal THs are necessary in the fetal brain before the maturation of the fetal thyroid gland. Interestingly, D2 and D3 are developmentally regulated in the brain (Bates et al., 1999; Escámez et al., 1999; Guadaño-Ferraz et al., 1999) as during the rat early postnatal development the maturational patterns of D2 and D3 activities reveal a progressive increase in D2 activity while D3 activity follows the opposite pattern (Kaplan and Yaskoski, 1981).

Regarding TH transporters there are no studies describing their spatiotemporal expression pattern during embryonic development in neither rats nor mice. There is a very complete study describing in detail the expression pattern of the main TH transporters in the mouse brain (Mct8, organic anion transporter family member 1c1 (Oatp1c1), L-type amino acid transporter 1, L-type amino acid transporter 2 and monocarboxylate transporter 10) from postnatal day 6 (P6) and this study revealed a precise mRNA expression pattern for each of the transporters which changed with age (Müller and Heuer, 2014). Other physiological studies have demonstrated that Mct8-deficient mice present a state of brain hyperthyroidism from E18 until P5 and that the combined inactivation of Mct8 and Lat2 suppresses this hyperthyroidism (Ferrara et al., 2013; Núñez et al., 2014) suggesting a crucial role for TH transporters in brain TH homeostasis. The dynamic expression of TH transporters in the brain as well as the functional implications of their deficiency highlights the relevance of TH transporters activity during fetal neurodevelopment and the consequent need to increase our understanding of their spatiotemporal expression pattern and function.

## Sources of Thyroid Hormones to The Rodent Brain

The main source of T3 in the brain is provided by the local conversion of T4 into T3 by D2 as, in the adult rat brain, 80% of nuclear bound T3 is formed locally from T4 (Crantz et al., 1982). Further studies in adult hypothyroid rats revealed that constant infusion of T3 at relatively low doses was enough to normalize T3 levels in peripheral tissues while much higher doses were necessary for the cerebral cortex (CCx) to reach normal values. On the other hand, when T4 was administered, T3 values were normalized in the CCx at lower doses than those necessary to restore T3 levels in other peripheral tissues. Furthermore, T3 content in the CCx remained within the physiological range regardless of the increase in the dose of the T4 administered (Escobar-Morreale et al., 1999). These studies revealed that T4 is the primary source of T3 in the brain and that local conversion of T4 into T3 is tightly regulated avoiding excess of T3. A previous study had proposed a close metabolic coupling between glial cells and neurons by which T4 would be taken up from the blood and the cerebrospinal fluid by astrocytes and tanycytes, where it would be deiodinated into T3 by D2 activity, and would finally be released for utilization by neurons (Guadaño-Ferraz et al., 1997). Years later, studies with transgenic mice validated and completed this model for T3 availability to neural cells (Ceballos et al., 2009; Freitas et al., 2010; Morte et al., 2010a). Currently, the model states that brain T3 has a double origin: a fraction is available directly from the circulation, and another is produced locally from T4 in the astrocytes by D2. To enter the brain, circulating T4 and T3 need to cross the brain barriers through specific TH transporters. Based on the transporters’ location the current model in rodents indicates that: (i) T3 and T4 cross the blood-brain barrier (BBB) through Mct8 (Dumitrescu et al., 2006; Trajkovic et al., 2007) into the extracellular fluid where they can directly reach the neural cells in the proximity of the blood vessels; and that (ii) T4, but not T3, crosses the BBB through the Oatp1c1 (Mayerl et al., 2014) directly into the astrocytes through their end-feet in contact with the blood vessels, and astrocytes produce additional T3 by D2 activity that can be then transported to the neurons (reviewed in Morte and Bernal, 2014).

For the postnatal and adult brain, studies in transgenic mice suggest that each of these two routes can contribute to at least 50% of the total pool of brain T3. D2-deficient mice present around a 50% decrease in the content of T3 in the CCx at P15 (Galton et al., 2007) and 40% decrease at 3 months of age (Bárez-López et al., 2014). Furthermore, Mct8-deficient mice also present a 50% reduction in the content of T3 in the CCx (Dumitrescu et al., 2006; Trajkovic et al., 2007). Together these data suggest that in the mice postnatal brain around 50% of the total brain T3 derives directly from T3 uptake from the circulation and another 50% is locally generated in the astrocytes from T4.

In contrast, studies in rats demonstrated that during fetal development the brain depends almost entirely on the T3 locally generated by D2. A pioneer study demonstrated that infusion of T4 to hypothyroid pregnant rats was sufficient to increase T3 content in the fetal brain to similar values of control fetuses while T3 concentrations increased very little in plasma and lung (Morreale de Escobar et al., 1988). Subsequent studies evidenced that the high doses of T3 administered to hypothyroid pregnant rats were able to cross the placenta but failed to normalize T3 content in the fetal brain and to induce the expression of T3-dependent genes. In contrast, physiological doses of T4 could cross both the placenta and the fetal brain barriers, normalizing fetal brain T3 concentrations by T4 deiodination and increasing neuronal gene expression (Calvo et al., 1990; Grijota-Martínez et al., 2011). The role of D2 activity in this process is further supported by D2 activity ontogeny studies that revealed significant D2 activity in rat fetal brain which highly increased prior to birth and was inversely regulated by increasing doses of T4 (Ruiz De Oña et al., 1988). The reason why the fetal brain is not permeable to T3 is not known and cannot be explained by lack of the Mct8 transporter as this is present at the BBB during fetal development (Grijota-Martínez et al., 2011). Likewise for the mouse, recent studies have demonstrated that maternal T4 is an essential source of TH in the developing mouse fetal brain and that T3 content is regulated through the conversion of T4 into T3 by D2 activity (Bárez-López et al., 2017b). However, the contribution of maternal T3 to the total fetal brain T3 content in mice still remains to be elucidated.

It still remains unclear whether maternal THs are necessary before the onset of the fetal thyroid gland. Some studies in rats indicated that maternal hypothyroidism leads to defects in the proliferation of some neuronal precursors that is usually completed by E12 (Narayanan and Narayanan, 1985), and to defects in the migration of some proliferating cells that normally finishes at E16–E17 (Lucio et al., 1997). In mice the treatment of pregnant dams with goitrogens from E13 to E16 led to a decrease in the T4 content in the fetal CCx and had an effect in the expression of a few T3-dependent genes (Dong et al., 2015). These findings might indicate that maternal THs are necessary before the fetal thyroid gland starts functioning, however, these defects could arise from maternal hypothyroidism side effects or from a direct effect of the goitrogens used to induce hypothyroidism. However one study in rats evidenced that maternal hypothyroxinemia derived from low iodine intake results in abnormal migration of neurons that is usually complete before the fetal thyroid function, strongly suggesting that maternal THs are necessary before the onset of the fetal thyroid gland (Lavado-Autric et al., 2003).

Another essential aspect that needs to be clarified is the contribution of maternal THs to the fetal brain under fetal euthyroid conditions. Studies in rats revealed that fetuses coming from thyroidectomized pregnant dams presented only a small decrease in the brain content of T4, with no changes in T3 content at perinatal stages (Berbel et al., 2010; Grijota-Martínez et al., 2011) and unaffected expression of T3-dependent genes (Morte et al., 2010b; Grijota-Martínez et al., 2011). Likewise, in another study, brain D2 activity of fetuses coming from thyroidectomized pregnant rats was not different from those coming from control dams (Ruiz De Oña et al., 1988). These findings in rats can be attributed to the progressive activity of the fetal thyroid gland indicating that fetal THs are the main regulators of TH-dependent fetal gene expression. Nevertheless, these results do not rule out a specific contribution from the mother. As the contribution of the fetal thyroid gland seems to be larger in rats than in mice (Bárez-López et al., 2017b), it should not be assumed that mice will follow the same pattern as rats. The difficulty of performing successful thyroidectomies in mice might hinder the assessment of the role of maternal THs in mice fetal brain under euthyroid fetal conditions.

## Brain Barriers and Thyroid Hormones

The passage of substances from the blood to the brain is regulated by the BBB and the blood-cerebrospinal fluid barrier (BCSFB). The BBB is formed by endothelial cells within CNS microvessels while the BCSFB is formed by a single epithelial cell layer in the choroid plexus and multiple epithelial cell layers in the arachnoid membrane (Johanson et al., 2011). These barriers tightly control the influx and efflux of molecules and ions at the blood-brain interface to meet neural cell needs and to protect the brain from toxins and pathogens (Obermeier et al., 2013). The severe alterations present in MCT8-deficient patients (Fu et al., 2013) revealed the relevance of TH transporters and the importance of TH transport across brain barriers (Ceballos et al., 2009; Iwayama et al., 2016; Vatine et al., 2017). Studies in the rat indicated that the contribution of the choroid plexus to the total brain TH content is about 18% (Chanoine et al., 1992) and, since the BBB surface area is much greater than that of the choroid plexus, the BBB has been considered the major pathway for TH entry into the brain (Dratman et al., 1991).

**Figure 2:**
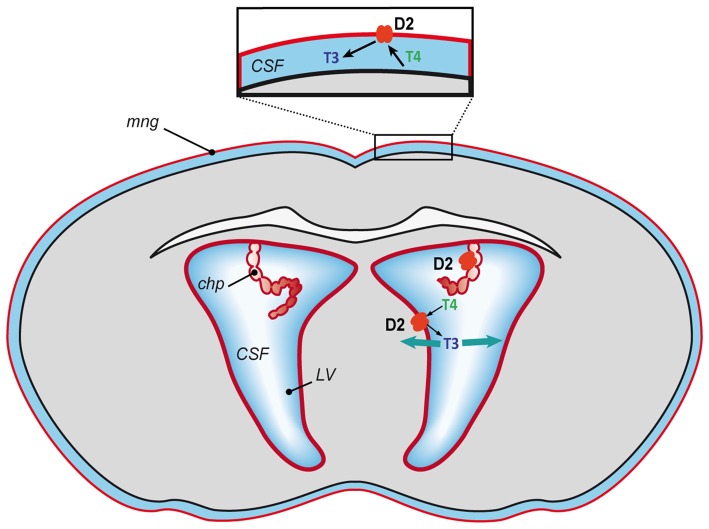
Proposed model of T3 availability to the mouse developing brain. T4 mostly of maternal origin reaches the brain, gets transported into the cerebrospinal fluid and converted into T3 by D2 (in red) at the blood-cerebrospinal fluid barrier (meninges and choroid plexus) and at the lateral ventricles. From the cerebrospinal fluid the T3 could spread throughout the brain and access the neural cells to exert its action. CFS, cerebrospinal fluid; chp, choroid plexus; LV, lateral ventricles and mng, meninges.

However, what is the situation in the fetal brain during the development of the BBB (Obermeier et al., 2013) and before the maturation of astrocytes (Daneman et al., 2010)? In a recent study we detected *Dio2* mRNA in the mouse brain in the meninges, the ependymal layer of the lateral ventricles and the choroid plexus at E15 and E18 (Bárez-López et al., 2017b). *Dio2* expression in the meninges, the ependymal layer of the lateral ventricles and the choroid plexus diminished by P3 and has not been detected in these structures in the adult brain neither in mice (Bárez-López et al., 2017a) nor rats (Wittmann et al., 2014). This indicates an important role of D2 at the BCSFB during fetal stages, especially since it has been suggested that the BCSFB could have a more important role during early stages of brain growth and development as the choroid plexus may have a greater transport capacity (Keep and Jones, 1990). Moreover, the volume of the lateral ventricles is larger at these stages in comparison to the rest of the brain (O’Rahilly and Müller, 2008). This suggests that the T4 transported through the choroid plexus could be deiodinated into T3 by D2 activity at the BCSFB and from the cerebrospinal fluid the T3 could reach the brain cells and exert and action at the genomic level (Figure 2). Furthermore, the generation of T3 by D2 at the meninges could be controlling the synthesis of retinoid acid (Gil-Ibáñez et al., 2014) regulating cortical neurogenesis (Siegenthaler et al., 2009). It has also been demonstrated that D2 deficiency during perinatal neurodevelopment leads to a state of brain hypothyroidism with reduced expression of T3-dependent genes (Bárez-López et al., 2017b). Altogether the current data indicate that conversion of T4 into T3 by D2 activity might be the only source of T3 during mouse brain development.

## Conclusion

Due to the crucial role of TH action during brain development and to consequences of TH deficit, it is essential to understand the physiological mechanisms that underlie T4 and T3 availability to the developing brain in order to obtain insight into pathological conditions. This will permit the development of appropriate treatments and establish timings to prevent, restore or at least palliate possible impairments, for example for MCT8-deficient patients. Studies in animal models have provided a great deal of information about the events underlying TH action during brain development, however, there are still many aspects that need to be elucidated. Among all the unresolved matters both in rat and mouse, a better understanding of the contribution of maternal THs to the fetal brain under euthyroid conditions, of the role of TH transporters during brain development and of the contribution of the BCSFB to TH brain availability would facilitate potential clinical applications in the future.

## Author Contributions

SB-L and AG-F have contributed in the drafting of the article and have revised it critically for important intellectual content and for the final approval of the submitted version. SB-L has designed the figures.

## Conflict of Interest Statement

The authors declare that the research was conducted in the absence of any commercial or financial relationships that could be construed as a potential conflict of interest.

## Abbreviations

BBB: blood brain barrier
BCSFB: blood-cerebrospinal fluid barrier
D2: deiodinase type-2
D3: deiodinases type-3
E: embryonic day
MCT8: monocarboxylate transporter 8
Oatp1c1: organic anion transporter family member 1C1
P: postnatal day
T3: 3,5,3′-triodo-L-thyronine
T4: 3,5,3′,5′-tetraiodo-L-thyronine
THs: thyroid hormones
TRs: thyroid hormone receptors.
